# Reviewer Summary for Journal of Arrhythmia

**DOI:** 10.1002/joa3.70374

**Published:** 2026-06-25

The Editorial Board members of the Journal of Arrhythmia are grateful to the following reviewers who provided their expertise and knowledge to the journal.

Yanagisawa, Satoshi

pinponstar@yahoo.co.jp

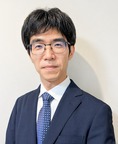

Fukaya, Hidehira

hidehira@med.kitasato-u.ac.jp

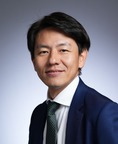

Ikeda, Yoshifumi

yooikeda@aol.com

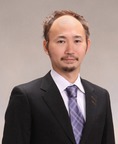

Tsutsui, Kenta

Yamashita, Seigo

Kondo, Yusuke

Nakahara, Shiro

Hachiya, Hitoshi

Kanzaki, Yasunori

Wakamiya, Akinori

Yamasaki, Hiro

Fukuzawa, Koji

Kataoka, Naoya

Noda, Takashi

Ogawa, Masahiro

Sasaki, Shingo

Arimoto, Takanori

Higuchi, Satoshi

Ishibashi, Kohei

Kato, Hiroyuki

Kimura, Masaomi

Matsunaga‐Lee, Yasuharu

Morita, Norishige

Ohe, Masatsugu

Ueda, Akiko

Aizawa, Yoshiyasu

Fukunaga, Masato

Hashimoto, Kenichi

Maruyama, Mitsunori

Masuda, Masaharu

Morishima, Itsuro

Nagase, Satoshi

Nakai, Toshiko

Nishii, Nobuhiro

Yoshida, Kentaro

Futyma, Piotr

Hayashi, Tatsuya

Imai, Katsuhiko

Inaba, Osamu

Jędrzejczyk‐Patej, Ewa

Kabutoya, Tomoyuki

Kawakami, Hiroshi

Kishihara, Jun

Kodani, Eitaro

Miyamoto, Koji

Miyazaki, Aya

Oginosawa, Yasushi

Otsubo, Toyokazu

Sekihara, Takayuki

Shimojo, Masafumi

Tanaka, Yasuaki

Watanabe, Masaya

Chen, Wei‐Ta

Enomoto, Yoshinari

Hasebe, Hideyuki

Ikee, Takashi

Kamakura, Tsukasa

Kondo, Hidekazu

Maruyama, Masahiro

Mukai, Yasushi

Nakamura, Kohki

Nakasuka, Kosuke

Naruse, Yoshihisa

Ogano, Michio

Sekiguchi, Yukio

Sobue, Yoshihiro

Suzuki, Shinya

Temma, Taro

Abe, Ichitaro

Fujimoto, Yuhi

Fukamizu, Seiji

Harada, Masahide

Hayashi, Kentaro

Inden, Yasuya

Kato, Takeshi

Kobori, Atsushi

Kohno, Ritsuko

Kumagai, Koji

Kurita, Takashi

Kuroki, Kenji

Kusa, Shigeki

Makiyama, Takeru

Mine, Takanao

Miyazaki, Shinsuke

Nakano, Makoto

Nakatani, Yosuke

Narita, Masataka

Nishiuchi, Suguru

Okubo, Kenji

Sairaku, Akinori

Sasaki, Wataru

Senoo, Keitaro

Suzuki, Tsugutoshi

Takami, Mitsuru

Takasugi, Nobuhiro

Takatsuki, Seiji

Tonegawa‐Kuji, Reina

Wada, Mitsuru

Watanabe, Ryuta

Yagishita, Atsuhiko

Yamagata, Kenichiro

Yeo, Colin

Yoshiga, Yasuhiro

Chang, Shih‐Lin

Kamada, Hiroyuki

Kaneko, Yoshiaki

Kato, Yoshiaki

Nagase, Takahiko

Okubo, Yousaku

Onishi, Yoshimi

Ono, Maki

Sano, Makoto

Shinohara, Tetsuji

Shiozawa, Tomoyuki

Sunaga, Akihiro

Takahashi, Naohiko

Tanabe, Yasuko

Ueda, Nobuhiko

Yakabe, Daisuke

Yamashita, Kennosuke

Yamauchi, Yasuteru

Yazaki, Yoshinao

Yodogawa, Kenji

Aizawa, Yoshifusa

Akao, Masaharu

An, Yoshimori

Aoki, Hisaaki

Ejima, Koichiro

Goto, Toshihiko

Hayashi, Hiroshi

Hayashi, Kenshi

Hojo, Rintaro

Kaneshiro, Takashi

Kawaji, Tetsuma

Kawakami, Tohru

Kawano, Daisuke

Komatsu, Yuki

Makimoto, Hisaki

Matsumoto, Kazuhisa

Miyanaga, Satoru

Mizukami, Akira

Mizutani, Yoshiaki

Murakami, Masato

Nabeshima, Taisuke

Nakajima, Kenzaburo

Nakamura, Keijiro

Nodera, Minoru

Nomura, Takehiro

Oikawa, Jun

Oka, Satoshi

Okumura, Yasuo

Preda, Alberto

Sato, Hiroyuki

Sato, Toshiaki

Shako, Daiki

Sonoda, Koichiro

Takenaka, Sou

Tanaka, Nobuaki

Tobiume, Takeshi

Uetake, Shunsuke

Yagishita, Daigo

Yamamoto, Teppei

Yokoshiki, Hisashi

Ali, Hussam

Ayabe, Kengo

Canpolat, Uğur

Chaturvedi, Vivek

Chishaki, Shoko

Choi, Young

Di Cori, Andrea

Egami, Yasuyuki

Fujino, Tadashi

Fujiu, Katsuhito

Fukuda, Masakazu

Furukawa, Toshiyuki

Furusho, Hiroshi

Hasegawa, Kanae

Hayashi, Hidemori

Hirata, Shu

Hu, Yu‐Feng

Imai, Yasushi

Imamura, Teruhiko

Inamura, Yukihiro

Irie, Tadanobu

Ishii, Yosuke

Kasagawa, Akira

Kawamura, Mitsuharu

Kim, Daehoon

Kowase, Shinya

Kuroda, Shunsuke

Lee, Pi‐Chang

Machino, Takeshi

Mori, Hitoshi

Morita, Junji

Muraji, Shota

Nagashima, Koichi

Nagashima, Michio

Nakamura, Tomofumi

Nitta, Takashi

Oka, Takafumi

Okamatsu, Hideharu

Osanai, Hiroyuki

Otsuka, Naoto

Rhee, Tae‐Min

Sagawa, Yuichiro

Sakamoto, Kazuo

Sasaki, Takeshi

Shehata, Islam

Shiga, Tsuyoshi

Shimeno, Kenji

Tabuchi, Haruna

Tao, Susumu

Yamaguchi, Takanori

Yasuhiro, Ogura

Amaya, Naoki

Ando, Monami

Arora, Shilpkumar

Chen, Guanglei

Ching, Chi Keong

Chinushi, Masaomi

Chung, Fa‐Po

Colluoglu, Inci Tugce

Fan, Xiaohan

Fujita, Shuhei

Fukunaga, Hiroshi

Furutani, Motoki

Goto, Koji

Hayashi, Meiso

Higuchi, Koji

Hori, Yuichi

Horie, Minoru

Inoue, Yuko

Isawa, Tsuyoshi

Jena, Nihar

Kaitani, Kazuaki

Kanazawa, Hisanori

Karimli, Emin

Kawamura, Iwanari

Kawashima, Hideyuki

Keida, Takehiko

Kim, Sung‐Hwan

Kinoshita, Toshio

Kiuchi, Kunihiko

Kobayashi, Takashi

Kodera, Satoshi

Kojima, Toshiya

Kuwahara, Taishi

Lau, Chu‐Pak

Lee, Adam

Liao, Ying‐Chieh

Lin, Lian‐Yu

Lin, Yenn‐Jiang

Maruyama, Toru

Minamiguchi, Hitoshi

Mito, Shinji

Miyagi, Yasuo

Mizobuchi, Masahiro

Morita, Hiroshi

Murase, Yosuke

Nakajima, Ikutaro

Nakamura, Kazufumi

Nakamura, Nobuhiro

Nakano, Masahiro

Narita, Yuji

Nishiyama, Nobuhiro

Ogiso, Sho

Ohno, Seiko

Oshima, Tsukasa

Ota, Shingo

Otsuki, Sou

Palamà, Zefferino

Park, Yae Min

Prana Jagannatha, Gusti Ngurah

Raharjo, Sunu

Satomi, Kazuhiro

Shinohara, Masaya

Shirai, Yasuhiro

Soejima, Kyoko

Sumiyoshi, Masataka

Suzuki, Makoto

Takahashi, Yoshihide

Takamiya, Tomomasa

Takigawa, Masateru

Tanno, Kaoru

Terata, Ken

Tokano, Takashi

Tokuda, Michifumi

Tomita, Hirofumi

Toyama, Hideko

Tsai, Chia‐Ti

Tsuboi, Ippei

Ueda, Shinichiro

Wakamatsu, Yuji

Watanabe, Atsuyuki

Wong, Christopher

Wong, Kam Cheong

Yagi, Tetsuo

Yamabe, Hiroshige

Yamada, Akira

Yamagami, Shintaro

Yamashiro, Kohei

Yamazaki, Masatoshi

Yokoyama, Yasuhiro

Yoshida, Akira

Yoshioka, Koichiro

Yuzawa, Hitomi

Zweiker, David

Abe, Yoshihisa

Abe, Yoshihsa

Anand, Abhinav

Arana‐Rueda, Eduardo

Ashikaga, Keiichi

Ashraf, Ali

Ashraf, Hamza

Boriani, Giuseppe

Çelik, Mustafa

Chan, Chin Pang

Chan, Ngai‐Yin

Chang, Ting‐Yung

Coll‐Vinent, Blanca

Danpanichkul, Pojsakorn

Deshmukh, Abhishek

Diaz, Juan Carlos

Dural, Muhammet

Emami, Mehrdad

Fujibayashi, Kosuke

Hamatani, Yasuhiro

Hara, Satoshi

Hendriks, Jeroen

Higa, Satoshi

Hsieh, Yu‐Cheng

Igarashi, Miyako

Ikeda, Takanori

Ishimura, Masayuki

J, Sivaraman

Jastrzebski, Marek

Jelavic, Marko Mornar

Joung, Boyoung

Kakuta, Takashi

Kamioka, Masashi

Kanaoka, Koshiro

Kasai, Yuhei

Kato, Kazuo

Kielbasa, Grzegorz

Kim, Joon Bum

Kim, Jun

Kim, Moon‐Hyun

Kim, Tae‐Hoon

Klein, Helmut

Koga, Msatoshi

Kojodjojo, Pipin

Koshy, Anoop N

Kurata, Masaaki

Kwon, Younghoon

Lau, Dennis

Lavalle, Carlo

Leal, Jenine

Lee, Justin

Lee, So‐Ryoung

Lever, Nigel

Liao, Jo‐Nan

Lin, Chin‐Yu

Lloyd, Michael

Lo, Li‐Wei

Luebbert, Jeffrey

Maeda, Akiko

Malik, Varun

Mari, Amino

Mascia, Giuseppe

Matsui, Yuko

Matsumoto, Hiroki

Matsuura, Haruka

Miki, Yuko

Miyajima, Keisuke

Miyauchi, Yasushi

Miyazaki, Yuichiro

Murata, Makoto

Nagai, Toshiyuki

Nagata, Yasutoshi

Nakagawa, Koji

Nakamura, Toshihiro

Nakao, Yoko M

Nishimori, Makoto

Nishimura, Takuro

Noda, Kazuki

Oda, Noboru

Odagiri, Fuminori

Okada, Atsushi

Okada, Ayako

Ono, Morio

Ozawa, Tomoya

Pachon, Jose Carlos

Pap, Robert

Rattanawong, Pattara

Rujirachun, Pongprueth

Sadek, Mouhannad M

Sakamoto, Shun‐Ichiro

Salinet, Joao

Sanders, Prashanthan

Segreti, L

Shim, Jaemin

Shimamoto, Keiko

Shiraishi, Hirokazu

Shroff, Jenesh

Sinan, Umit Yasar

Soeki, Takeshi

Sommer, Philipp

Sonaglioni, Andrea

Srinivasan, Neil

Sterliński, Maciej

Stiles, Martin

Straube, Florian

Sumitomo, Naokata

Suzuki, Yasushi

Syed, Faisal F. F.

Takagi, Masahiko

Takahashi, Kenta

Takahashi, Masao

Takaya, Yoichi

Takemoto, Masao

Takeuchi, Daiji

Tanaka, Kanta

Tanimoto, Kojiro

Tokutake, Ken‐Ichi

Tomii, Naoki

Traykov, Vassil

Tseveendee, Saruul

Uehara, Hiroki

Ueno, Yuji

Yamada, Shinya

Yano, Kensuke

Yasuoka, Ryobun

Yazaki, Kyoichiro

Young, Glenn

Zucchelli, Giulio

